# The impact on colostrum oxidative stress, cytokines, and immune cells composition after SARS-CoV-2 infection during pregnancy

**DOI:** 10.3389/fimmu.2022.1031248

**Published:** 2022-12-15

**Authors:** Nayara Gomes Graciliano, Micaely Cristinados Santos Tenório, Marilene Brandão Tenório Fragoso, Fabiana Andréa Moura, Rayane Martins Botelho, Eloiza Lopes Lira Tanabe, Karen Steponavicius Cruz Borbely, Alexandre Urban Borbely, Alane Cabral Menezes Oliveira, Marília Oliveira Fonseca Goulart

**Affiliations:** ^1^ Institute of Biological and Health Sciences, Federal University of Alagoas, Maceio, Alagoas, Brazil; ^2^ Institute of Chemistry and Biotechnology, Federal University of Alagoas, Maceio, Alagoas, Brazil; ^3^ College of Nutrition, Federal University of Alagoas, Maceio, Alagoas, Brazil; ^4^ National Institute of Science and Technology in Bioanalytics (INCT-Bio), Campinas, Sao Paulo, Brazil

**Keywords:** antibodies, breastfeeding, COVID-19, human milk, immunoglobulins, IgA, passive immunity

## Abstract

**Background:**

Limited data are available regarding the differences between immunological, biochemical, and cellular contents of human colostrum following maternal infection during pregnancy with coronavirus 2 disease (COVID-19).

**Objective:**

To investigate whether maternal COVID-19 infection may affect immunological, biochemical, and cellular contents of human colostrum.

**Methods:**

Using a case-control study design, we collected colostrum from 14 lactating women with a previous diagnosis of COVID-19 during pregnancy and 12 without a clear diagnosis during September 2020 to May 2021. Colostrum samples were analysed for some enzymes and non-enzymatic oxidative stress markers (SOD, CAT, GPx, MDA, GSH, GSSG, H_2_O_2_, MPO) and for IL-1β, IL-6, tumour necrosis factor (TNF)-α, protein induced by interferon gamma (IP)-10, IL-8, IFN-λ1, IL12p70, IFN-α2, IFN-λ2/3, granulocyte macrophage colony stimulating factor (GM-CSF), IFN-β, IL-10 and IFN-γ, along with IgA and IgG for the SARS-CoV-2 S protein. We perform immunophenotyping to assess the frequency of different cell types in the colostrum.

**Results:**

Colostrum from the COVID-19 symptomatic group in pregnancy contained reduced levels of H_2_O_2_, IFN-α2, and GM-CSF. This group had higher levels of GSH, and both NK cell subtypes CD3^-^CD56^bright^CD16^-^CD27^+^IFN-γ^+^ and CD3^-^CD56^dim^CD16^+^CD27^-^ were also increased.

**Conclusion:**

The present results reinforce the protective role of colostrum even in the case of mild SARS-Cov-2 infection, in addition to demonstrating how adaptive the composition of colostrum is after infections. It also supports the recommendation to encourage lactating women to continue breastfeeding after COVID-19 illness.

## Introduction

Coronavirus disease 2019 (COVID-19), represented by the severe acute respiratory syndrome caused by the SARS-CoV-2 virus, has spread worldwide (1). Patients with COVID-19 have different clinical patterns, such as asymptomatic, mild, moderate, severe, and up to critical types (2). During pregnancy, COVID-19 has been linked with an increased risk of preeclampsia, preterm birth, and stillbirth (3), but its repercussions on breast colostrum composition after asymptomatic and mild COVID-19 during pregnancy were not yet described.

The colostrum is the first breast secretion after childbirth. It has a yellow colour, sticky consistency, and a different composition from the mature milk, being rich mainly in beta-carotenes, immunoglobulin (Ig)A, fat-soluble vitamins, lactoferrin, sodium, and zinc, generating numerous benefits for the child, ranging from protection against pathogens to intestinal colonization (4). Additionally, the colostrum contains a plethora of immunological, biochemical, and cellular contents, which can cause significant modifications to the newborn immunity and susceptibility to infection (5). The immunity is, passively, transferred from colostrum to the newborn, especially due to the number of lymphocytes, macrophages, and their properties related to immune protection (6). In this context, given that COVID-19 vaccines are not yet approved for neonates or children younger than six months old, the passive immunity conferred by breastfeeding is unique to providing immunological defence to such a population (7).

To date, there is no robust scientific evidence that SARS-CoV-2 is transmitted through breastfeeding. Despite few reports on colostrum changes after COVID-19 infection, several blood components have already been identified during COVID-19. Among them, high serum levels of malondialdehyde (MDA), higher serum activity of catalase (CAT), of superoxide dismutase (SOD), in addition to severe glutathione (GSH) deficiency have been described (8, 9). Alterations in neutrophils, lymphocytes, natural killer (NK) cells, and thrombocyte counts, increased levels of coagulation parameters, ferritin, C-reactive protein, cytokines, and chemokines have also been reported during SARS-CoV-2 infection (10–12).

Likewise, colostrum composition changes could reflect alterations caused by COVID-19 in the mother. It is already known that colostrum and breast milk produced by women with COVID-19 contains actual immunoglobulin A (IgA), targeting the S glycoprotein receptor binding domain in the first month following infection (13–15). In addition, colostrum from symptomatic women with COVID-19 had been described to present higher levels of interferon (IFN)-γ, interleukin (IL)-4, IL-6, and IL-12 compared to asymptomatic lactating women with COVID-19 (15). Nevertheless, the repercussions of pregnancy and previous infection by SARS-CoV-2 to the colostrum composition remains unveiled. As such, the focus of the present work was to investigate alterations in colostrum oxidative stress, cytokines, and immune cells composition in asymptomatic breastfeeding women who developed mild COVID-19 symptoms, during pregnancy.

## Methodology

### Subject recruitment

A case-control study with healthy breastfeeding women that were SARS-CoV-2 infected during pregnancy. The recruitment occurred from September 2020 to May 2021. The women were divided into two groups: the symptomatic (n = 14), which tested positive for SARS-CoV-2 during pregnancy (any trimester) and presented mild symptoms, such as low fever, cough, shortness of breath, sore throat, and loss of taste and/or sense of smell, without the need for hospitalization; and the asymptomatic (n = 12), with no symptoms of COVID-19. The colostrum of all patients from both groups was IgA positive for the S protein of SARS-CoV-2 by ELISA (Euroimmun, Germany). It was assumed herein that IgA-positive women were exposed to SARS-CoV-2, since they were all unvaccinated. The ELISA kit manufacturer only found cross-reactivity with the S protein for SARS-CoV, which never circulated in Brazil (16). Other inclusion criteria for all groups were no current COVID-19 and/or neither infectious, autoimmune diseases, fever (current or recent), along with no gemelar pregnancy.

The Ethical Committee approved the study for human subjects (n. 36006920.8.0000.5013), and all women signed a free and informed consent. The recruitment occurred in three hospitals in Maceio-Alagoas, Brazil: University Hospital Professor Alberto Antunes (HUPAA), Santa Monica Maternity School (MESM), and Santo Antonio General Hospital. Socioeconomic, demographical, anthropometrical, and clinical data from the participants and their newborns were obtained through questionnaire and clinical records.

### Characterization of lactating women and their newborns

Demographic, socioeconomic, obstetric, and nutritional status data of lactating women were used to characterize the study population. The groups were classified according to age (≤ 19 years: adolescents; 20−34 years: average age; and ≥ 35 years: advanced age), self-declaration of black race (yes or no), education (≤ 9 years; 10−12 years; and ≥ 13 years of study), monthly family income (≤ 1 minimum wage; > 1 minimum wage), beneficiary of government social programs (yes or no), primiparous (yes or no), complications during pregnancy (yes or no), and mode of delivery (normal or caesarean).

The assessment of maternal nutritional status was performed, through height and current weight, measured with a digital scale with a stadiometer to calculate the Body Mass Index (BMI), and classified according to Atalah et al. (1997) (17). The newborn data were obtained by consulting the medical records and declaring live births. Their characterizations were based on the INTERGROWTH-21^st^ curves (18) by classifying weight and length at birth in percentiles. For the weight variable, the following was adopted: less than 10^th^ percentile—small for gestational age (SGA); between the 10^th^ and 90^th^ percentiles—adequate for gestational age (SUGA); and greater than 90^th^ percentile—large for gestational age (LGA). Length at birth was classified as low, adequate, and high, according to the same standards as weight. Gestational age at delivery was classified as an early term (37 0/7 weeks gestation to 38 6/7 weeks gestation), full-term (39 0/7 weeks gestation to 40 6/7 weeks gestation), late-term (41 0/7 weeks to 41 6/7 weeks of gestation) and post-term (42 0/7 weeks of gestation), according to the recommendation of the American College of Obstetricians and Gynecologists (19). The ratio of the chest and head circumference (CC/HC) was used to complement the information on the nutritional status of the newborn, being classified as adequate when the value was equal to 1 (20). Apgar scores were evaluated at the 1^st^ and 5^th^ minutes of life and classified as adequate when ≥ 7 (21).

### Sample collection and storage

**Figure 1:**
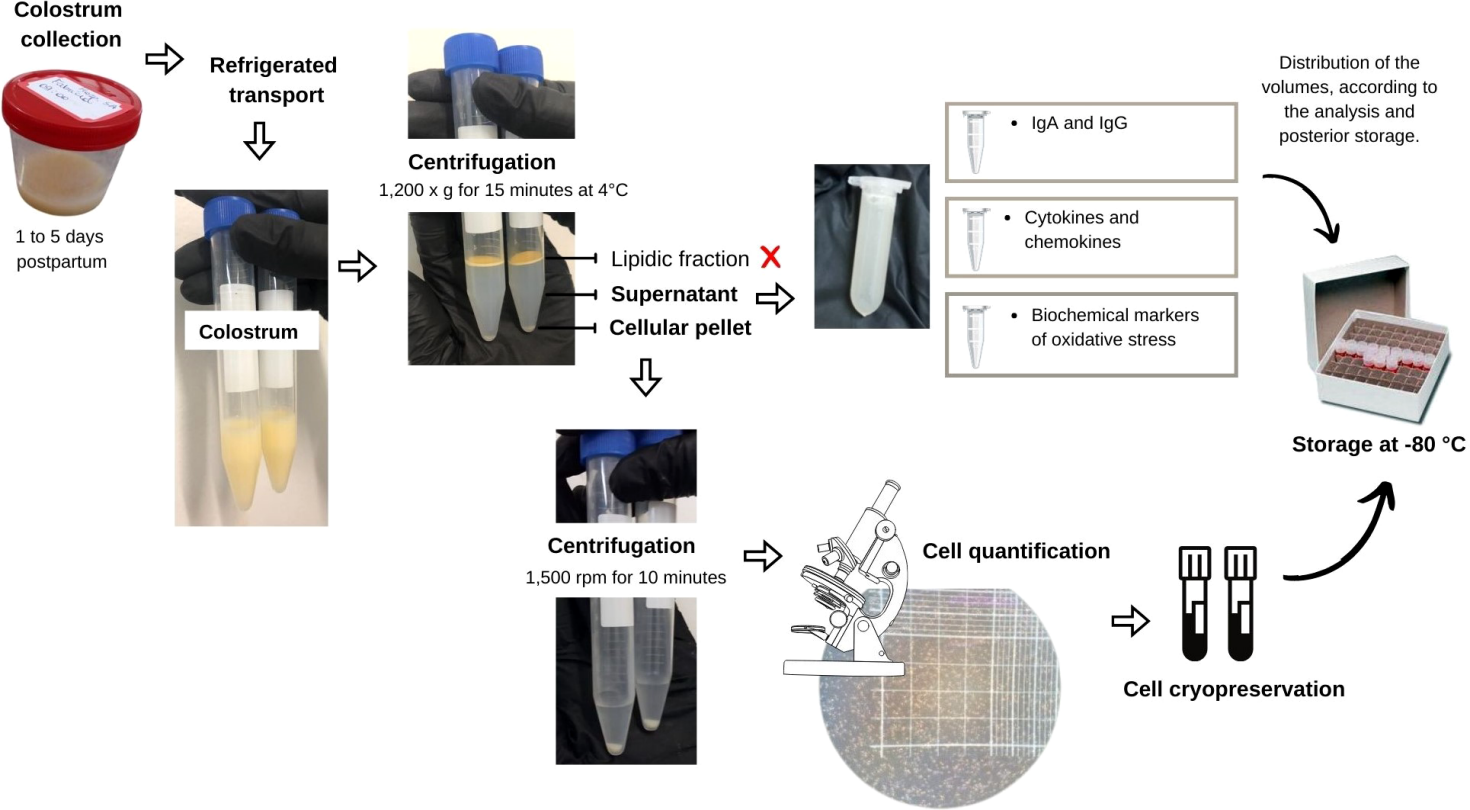
Flowchart representing the step of collection and preparation of colostrum for storage.

Sample collection was standardized and obtained by trained professionals duly equipped with personal protective equipment. A range from 4 mL to 10 mL of colostrum was obtained from one or both breasts, from 8 a.m. to 11 a.m., between 1 to 5 days postpartum. Briefly, the professional’s hands and women’s breasts were soap-washed, followed by 70% ethanol cleaning. The participants also received a triple protection surgical mask and a disposable cap. The professionals also used sterile surgical gloves, when handling the breasts to avoid colostrum contamination. The samples were collected in sterile containers and promptly stored, in ice, for fast transport to the Cell Biology Laboratory at the Federal University of Alagoas (UFAL), Brazil. The samples were centrifuged at 1,200 *g* for 15 min at 4°C, and the upper lipid fraction was carefully removed with a cotton swab. The translucid liquid supernatant was removed and stored at – 80°C for further cytokines and oxidative stress markers assessment. All analyzes were performed within 10 months after collection to minimize the loss of immunological properties (22). The cellular pellet was PBS-washed thoroughly, with further centrifugation at 1,500 rpm for 10 min. The total cell amount was counted in a Neubauer chamber under a light microscope. The samples were stored until further use at -80°C in cryopreservative solution: foetal bovine serum (FBS) and dimethyl sulfoxide (DMSO), at 9:1 proportion (**Figure 1**).

### IgA and IgG ELISA analyses

The samples were analysed for IgA and IgG for the SARS-CoV-2 S protein (Euroimmun, Lübeck, Germany), following the manufacturer’s instructions. Briefly, samples were thawed, centrifuged at 800 *g* for 15 min at room temperature, and tested in separate assays measuring IgA and IgG. Plates were incubated at 37°C for 1h, washed, and conjugated with peroxidase-labelled anti-human antibodies for 30 min at 37°C. The chromogen/substrate solution was added for an additional 30 min, followed by the addition of the stop solution. Photometric measures were acquired at 450 nm wavelength with a plate reader (Merck, Berlin, Germany).

### Biochemical markers of oxidative stress measurements

Total protein levels in colostrum samples were quantified by the colorimetric method of Bradford (1976) (23) to normalize the results of subsequent analyses by protein content. Superoxide dismutase (SOD) was analysed using the SOD Assay Kit – WST (Sigma Aldrich, St. Louis, MO, USA), following the manufacturer’s instructions, with the spectrophotometer reading performed at 450 nm and the activity expressed in U mg protein^-1^. Hydrogen peroxide (H_2_O_2_) was measured, according to a modified colorimetric method by Pick and Keisari (1980) (24), and described in µmol mg protein^-1^, following the spectrophotometer reading at 610 nm.

CAT was measured according to Paton et al. (25), previously described by Aebi (26). The method consists of monitoring the rate of decomposition of H_2_O_2_ in a spectrophotometer at 240 nm, with readings taken every 15 seconds for 5 min, with results expressed in U of CAT mg protein^-1^. A CAT unit is defined as the amount of enzyme needed to decompose, at 37°C, 1 μmol min^-1^ of H_2_O_2_.

The GSH and oxidized glutathione (GSSG) levels were determined according to a modified technique initially described by Tipple and Rogers (2012) (27) and expressed in pmol mg protein^-1^. The ratio (GSH/GSSG) was later calculated by applying the formula: Total glutathione = GSH - (2 x GSSG).

The total activity of glutathione peroxidase (GPx) was measured using the technique adapted from Flohé and Gunzler (1984) (28), with the addition of glutathione reductase (GR), GSH, and after, nicotinamide adenine dinucleotide phosphate (NADPH) and *tert*-butyl hydroperoxide (*t*-BuOOH). One unit of GPx corresponds to the amount of enzyme capable of catalysing the oxidation of 1 μmol of NADPH to NADP^+^ in 1 min. The result was expressed as nmol of NADPH consumed min mg protein^-1^.

MDA levels were evaluated using high-performance-liquid-chromatography (HPLC), measuring the peak height according to the method proposed by Vickie et al. (1990) (29) and expressed as nM mg protein^-1^.

The activity of the inflammation enzymatic biomarker myeloperoxidase (MPO) was quantified by the method proposed by Bradley et al. (1982) (30), with modifications, in which a unit of MPO is characterized as the amount of H_2_O_2_ decomposed per min. Results were expressed as U mg protein^-1^.

Reagents and chemicals were purchased from Sigma-Aldrich Chemicals (St. Louis, MO, USA). The analyzis was performed using HPLC (LC-20 AT-Prominence, Shimadzu) coupled to a UV detector (Shimadzu, serial no. L201550) and a Sanyo VIP Series freezer. The spectrofluorometer used was manufactured by Thermo Fisher Scientific^®^ (Multiskan).

### Cytokines and chemokines analyses

The cytokines and chemokines were detected with a Legendplex™ human anti-virus response panel (13-plex—Cat. 740390, Lot B310340, BioLegend, UK), for IL-1β, IL-6, tumour necrosis factor (TNF)-α, interferon gamma-induced protein (IP)-10, IL-8, IFN-λ1, IL12p70, IFN-α2, IFN-λ2/3, granulocyte-macrophage colony-stimulating factor (GM-CSF), IFN-β, IL-10, and IFN-γ, following the manufacturer instructions. Results were acquired in a FACS Canto II^®^ (BD Biosciences, Franklin Lakes, NJ, EUA), using the FACS Diva software (BD Biosciences). The results were analysed with the software Legendplex v.8.0 (BioLegend).

### Immune phenotyping

Cells were thawed and cultured in DMEM/F12 media with 20% FBS and 1% streptomycin and penicillin (Merck-Sigma-Aldrich, St. Louis, MO, USA) at 37 °C for 4 h. Then, cells were washed in PBS-BSA 0.5% with 0.02% sodium azide, centrifuged at 1,500 rpm for 5 min at 4 °C, counted again, and separated as 2 × 10^5^ cells per tube. Afterward, cells were stained with antibodies (**Table 1**). Briefly, cells were incubated with each antibody for 20 min at 4 °C, washed with PBS-BSA 0.5% with 0.02% sodium azide, and fixed with BD Cytofix/Cytoperm™ (BD Biosciences). After fixation, the cells were washed with BD Perm/Wash™ (BD Biosciences), centrifuged, and resuspended in PBS-BSA 0.5% with 0.02% sodium azide for measurements in a FACS Canto II^®^, using the FACS Diva software. Gating and data analyzis were performed using the software FlowJo^®^ X (BD Biosciences).

**Table 1:** List of antibodies used for immunophenotyping.

Antibody	Isotype	Clone	Catalog, Manufacturer
Anti-CD3-FITC	Mouse IgG1, κ	UCHT1	300406, Biolegend
Anti-CD3-PERCPCy5.5	Mouse IgG1, κ	UCHT1	560835, BD Biosciences
Anti-CD4-PERCPCy5.5	Mouse IgG2b, κ	OKT4	317428, Biolegend
Anti-CD8-FITC	Mouse IgG1, κ	RPA-T8	555366, BD Biosciences
Anti-CD16-PE	Mouse IgG1, κ	B73.1	561313, BD Biosciences
Anti-CD19-PE	Mouse IgG1, κ	HIB19	302208, Biolegend
Anti-CD27-PECy7	Mouse IgG1, κ	M-T271	560609, BD Biosciences
Anti-CD45RO-APC	Mouse IgG2a, κ	UCHL1	559865, BD Biosciences
Anti-CD56-APC	Mouse IgG1, κ	HCD56	318310, Biolegend
Anti-CD62L-PECy7	Mouse IgG1, κ	DREG-56	304822, Biolegend
Anti-CD138-APC	Mouse IgG1, κ	MI15	347193, BD Biosciences
Anti-Tbet-PE	Mouse IgG1, κ	O4-46	561268, BD Biosciences
Anti-RORγt-APC	Mouse IgG2b, κ	Q21-559	563620, BD Biosciences
Anti-GATA3-PECy7	Mouse IgG1, κ	L50-823	560405, BD Biosciences
Anti-IFN-γ	Mouse IgG2b, κ	IFNG/466	NBP2-53332, Novus Biologicals, USA
Anti-IL-17A-PE	Mouse IgG1, κ	SCPL1262	560436, BD Biosciences
Zenon™ anti-mouse IgG2b-Alexa 488	Goat Fab fragments	–	Z25202, Thermo Fisher Scientific, San Diego, CA, USA

The immunophenotyping was performed to assess the frequency of the following cells: CD3-CD19^-^CD138^+^CD27^++^, CD3^-^CD19^+^CD138^-^CD27^+^, CD3^-^CD19^+^CD138^-^CD27^-^, CD3^+^CD4^+^CD45RO^-^CD62L^+^, CD3^+^CD8^+^CD45RO^-^CD62L^+^, CD3^+^CD4^+^CD45RO^+^CD62L^+^, CD3^+^CD8^+^CD45RO^+^CD62L^+^, CD3^+^CD4^+^CD45RO^+^CD62L^-^, CD3^+^CD8^+^CD45RO^+^CD62L^-^, CD3^+^CD4^+^CD45RO^-^CD62L^-^, CD3^+^CD8^+^CD45RO^-^CD62L^-^, CD3^-^CD56^bright^CD16^-^CD27^+^IFN-γ^+^, CD3^-^CD56^dim^CD16^+^CD27^-^, CD3^+^CD4^+^Tbet^+^, CD3^+^CD4^+^RORγt^+^IL17A+, CD3^+^CD4^+^GATA3^+^, CD3^-^CD4^-^Tbet^+^, CD3^-^CD4^-^RORγt^+^, and CD3^-^CD4^-^GATA3^+^.

### Statistical analyses

Descriptive statistics were used to characterize the symptomatic and asymptomatic breastfeeding groups. Categorical data were presented by frequencies and compared using the chi-square test. The Shapiro-Wilk test was used to assess the normality of data distribution. Differences between groups were examined using Student’s t test or the nonparametric Mann-Whitney test, depending on their distribution. All analyses were performed using Stata/MP 13.0 (https://www.stata.com ) and Graph Pad Prism 6 (GraphPad Software Inc., San Diego, CA, USA) software, considering *p* < 0.05 as the level of statistical significance.

## Results

### Characterization of lactating women and their newborns

The study was conducted with 26 lactating women aged 18 to 37 years (28.3 years ± 5.3). The main characteristics of the study population are listed (**Table 2**). There were no significant differences between the groups. In the symptomatic group, the manifestation of COVID-19 symptoms occurred predominantly in the first and third trimesters of pregnancy (35.7%), with 28.6% of pregnant women presenting the infection in the second trimester. The neonatal characteristics are presented (**Table 3**). In general, the neonates were born late-term, with good weight and length for their gestational age, and without complications.

**Table 2:** Demographic, socioeconomic, obstetric and nutritional status characteristics of symptomatic (n=14) and asymptomatic (n=12) for Covid-19 of lactating women, during pregnancy.

	Symptomatic		Asymptomatic		*P*-value
	n	%	N	%	
Age (years)					
≤ 19	0	0	1	8.3	0.386
20-34	11	78.6	10	83.4	
≥ 35	3	21.4	1	8.3	
Self-declaration of black race					
Yes	2	14.3	1	8.3	0.636
No	12	85.7	11	91.7	
Education (years)					
≤ 9	3	21.4	4	33.3	0.666
10-12	4	28.6	4	33.3	
≥ 13	7	50.0	4	33.3	
Monthly family income (minimum wage)					
≤ 1	0	0	2	16.7	0.112
> 1	14	100	10	83.3	
Government program beneficiary					
Yes	7	50.0	7	58.3	0.671
No	7	50.0	5	41.7	
Primiparous					
Yes	5	35.7	2	16.7	0.275
No	9	64.3	10	83.3	
Intercurrences in pregnancy					
Yes	8	57.1	5	41.7	0.431
No	6	42.9	7	58.3	
Mode of delivery					
Normal	5	35.7	1	8.3	0.099
Cesarean	9	64.3	11	91.7	
Gestational BMI					
Low weight	0	0	1	8.3	0.323
Eutrophy	6	42.9	2	16.7	
Overweight	2	14.3	1	8.3	
Obesity	6	42.8	8	66.7	

BMI, Body mass index. Chi-square test, p < 0.05.

**Table 3:** Neonatal characteristics of symptomatic (n=14) and asymptomatic (n=12) for Covid-19 of lactating women, during pregnancy.

	Symptomatic		Asymptomatic	
	n	%	N	%
Sex				
Men	8	57.1	5	41.7
Women	6	42.9	7	58.3
Gestational age at birth				
Early term	5	35.7	4	33.3
Full term	0	0	1	8.3
Late term	8	57.2	5	41.7
Postterm	1	7.1	2	16.7
Birth weight				
SGA	0	0	0	0
AGA	11	78.6	11	91.7
LGA	3	21.4	1	8.3
Length at birth				
Low	1	7.1	1	8.3
Adequate	12	85.7	11	91.7
High	1	7.1	0	0
Apgar 1st minute				
≤6	0	0	1	8.3
≥7	14	100.0	11	91.7
Apgar 5th minute				
≤6	0	0	0	0
≥7	14	100.0	12	100.0
CC/HC ratio				
Adequate	12	92.3	11	91.7
Inadequate	1	7.7	1	8.3
No information	1			

SGA, Small for gestational age; AGA, Appropriate for gestational age; LGA, Large for gestational age; CC, Chest circumference; HC, Head circumference.

### Measurement of biochemical markers of oxidative stress

**Figure 2:**
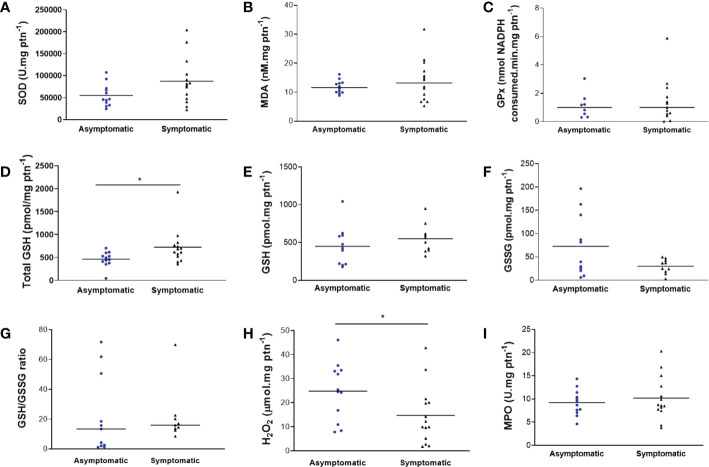
Biomarkers of oxidative stress in colostrum of symptomatic (n=14) and asymptomatic (n=12) for SARS-CoV-2 of lactating women, during pregnancy. **(A)** Concentration of SOD (U mg protein^-1^). **(B)** Concentration of MDA (nM mg protein^-1^). **(C)** Concentration of GPx (nmol NADPH consumed min mg protein^-1^). **(D)** Concentration of total GSH (pmol mg protein^-1^) (*p = 0.0202). **(E)** Concentration of GSH (pmol mg protein^-1^). **(F)** Concentration of GSSG (pmol mg protein^-1^). **(G)** Concentration of GSH/GSSG ratio. **(H)** Concentration of H_2_O_2_ (µmol mg protein^-1^) (*p = 0.0422). **(I)** Concentration of MPO (U mg protein^-1^). Data are presented as median and 95% CI. *indicates a statistical difference, comparing Asymptomatic *versus* Symptomatic. Mann–Whitney test: *p* < 0.05.

In the colostrum of the symptomatic group, there was an increase in total GSH (653.9 ± 385.2 μmol mg protein^-1^ and 475.4 ± 167.3 μmol mg protein^-1^; *p* = 0.0202) (**Figure 2D**) and a reduction in H_2_O_2_ (14.68 ± 12.08 μmol mg protein^-1^ and 24.84 ± 11.97 μmol mg protein^-1^; *p* = 0.0422) (**Figure 2H**) compared to the asymptomatic group. The graphical analyzis of **Figure 2F** also suggests a trend towards a reduction in GSSG in the symptomatic group, but without statistical significance (29.79 ± 15.40 pmol mg protein^-1^ and 72.66 ± 66.84 pmol mg protein^-1^; *p* = 0.0628). For the other biomarkers, the results were not significantly different (**Figures 2A–I**). No CAT activity was found in the samples. The analyzis was repeated for confirmation.

### Cytokines measurements

**Figure 3:**
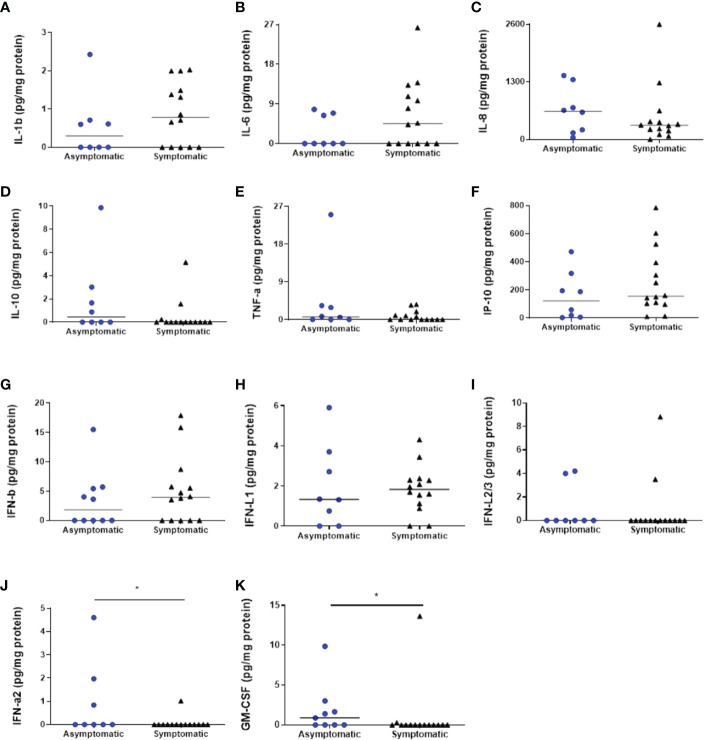
Cytokines’ analyzes in the colostrum of symptomatic (n=14) and asymptomatic (n=8) for Covid-19 of lactating women, during pregnancy. **(A)** Concentration of IL-1β (pg mg protein^-1^). **(B)** Concentration of IL-6 (pg mg protein^-1^). **(C)** Concentration of IL-8 (pg mg protein^-1^). **(D)** Concentration of IL-10 (pg mg protein^-1^). **(E)** Concentration of TNF-α (pg mg protein^-1^). **(F)** Concentration of IP-10 (pg mg protein^-1^). **(G)** Concentration of IFN-β (pg mg protein^-1^). **(H)** Concentration of IFN-λ1 (pg mg protein^-1^). **(I)** Concentration of IFN-λ2/3 (pg mg protein^-1^). **(J)** Concentration of IFN-α2 (pg mg protein^-1^) (*p = 0.0423). **(K)** Concentration of GM-CSF (mg protein^-1^) (*p = 0.0243). Data are presented as median and 95% CI. Mann–Whitney test: *p* < 0.05.

Several cytokines were detected in our samples. Nevertheless, IL-12p70 and IFN-γ had undetectable levels in both groups. The majority of the measured cytokines were detected in both groups, such as IL-1β, IL-6, IL-8, IL-10, TNF-α, IP-10, IFN-β, IFN-λ1, and IFN-λ2/3, although their levels were unchanged in all analysed conditions (**Figures 3A–I**). Regarding IFN-α2, the symptomatic group had decreased levels in comparison to the asymptomatic group (respectively, 0.07286 ± 0.07286 pg mg protein^-1^ and 0.9263 ± 0.5807 pg mg protein^-1^; *p* = 0.0423) (**Figure 3J**). The same pattern was observed with GM-CSF, with reduced levels in the symptomatic group compared to the asymptomatic group (respectively, 0.9943 ± 0.9730 pg mg protein^-1^ and 1.870 ± 1.057 pg mg protein^-1^; *p* = 0.0243) (**Figure 3K**).

### IgA and IgG analysis

**Figure 4:**
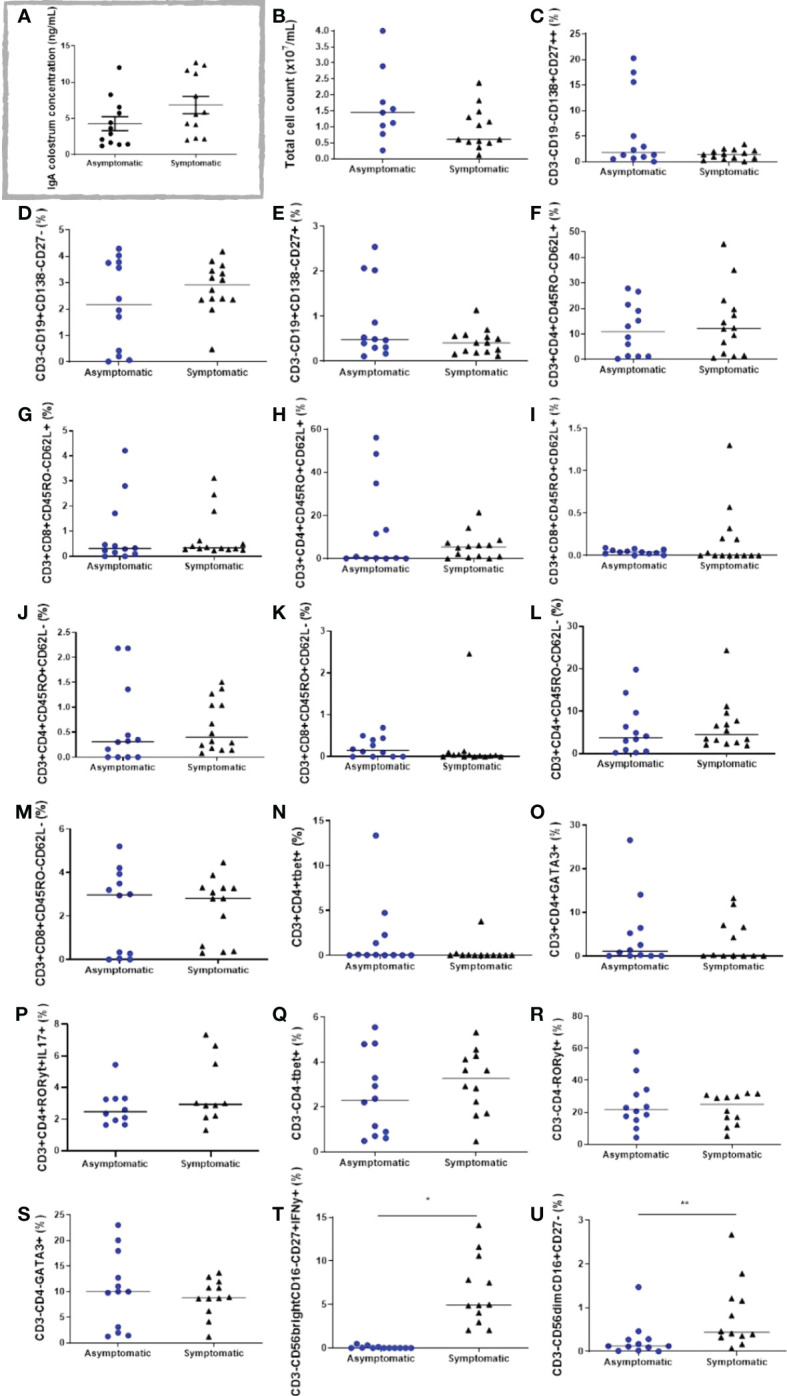
Cell populations in the colostrum of symptomatic (n=14) and asymptomatic (n=12) for Covid-19 of lactating women, during pregnancy. **(A)** IgA colostrum concentration (ng mL^-1^). **(B)** Total cell count (x10^7^ mL^-1^). **(C)** Percentage of cells CD3-CD19^-^CD138^+^CD27^++^. **(D)** Percentage of cells CD3^-^CD19^+^CD138^-^CD27^-^. **(E)** Percentage of cells CD3^-^CD19^+^CD138^-^CD27^+^. **(F)** Percentage of cells CD3^+^CD4^+^CD45RO^-^CD62L^+^. **(G)** Percentage of cells CD3^+^CD8^+^CD45RO^-^CD62L^+^. **(H)** Percentage of cells CD3^+^CD4^+^CD45RO^+^CD62L^+^. **(I)** Percentage of cells D3^+^CD8^+^CD45RO^+^CD62L^+^. **(J)** Percentage of cells CD3^+^CD4^+^CD45RO^+^CD62L^-^. **(L)** Percentage of cells CD3^+^CD8^+^CD45RO^+^CD62L^-^. **(K)** Percentage of cells CD3^+^CD4^+^CD45RO^-^CD62L^-^. **(M)** Percentage of cells CD3^+^CD8^+^CD45RO^-^CD62L^-^. **(N)** Percentage of cells CD3^+^CD4^+^Tbet^+^. **(O)** Percentage of cells CD3^+^CD4^+^GATA3^+^. **(P)** Percentage of cells CD3^+^CD4^+^RORγt^+^IL17A^+^. **(Q)** Percentage of cells CD3^-^CD4^-^Tbet^+^. **(R)** Percentage of cells CD3^-^CD4^-^RORγt^+^. **(S)** Percentage of cells CD3^-^CD4^-^GATA3^+^. **(T)** Percentage of cells CD3^-^CD56^bright^CD16^-^CD27^+^IFN-γ^+^. **(U)** Percentage of cells CD3^-^CD56^dim^CD16^+^CD27^-^. Data are presented as median and 95% CI. Mann–Whitney test: *p* < 0.05. *p < 0.000. ** *p* = 0.0093.

The colostrum was analysed for IgG and IgA specific for the SARS-CoV-2 S protein, as described before. No IgG whatsoever was found (data not shown). Nevertheless, IgA was present in all samples, without significant differences among the groups (**Figure 4A**).

### Immunophenotyping

The immunophenotypes from different lymphoid cells in the colostrum of both groups were analysed by flow cytometry. In general, total cell count and CD3^+^ cells tended to be reduced in the symptomatic group (**Figure 4B**).

**Figure 5:**
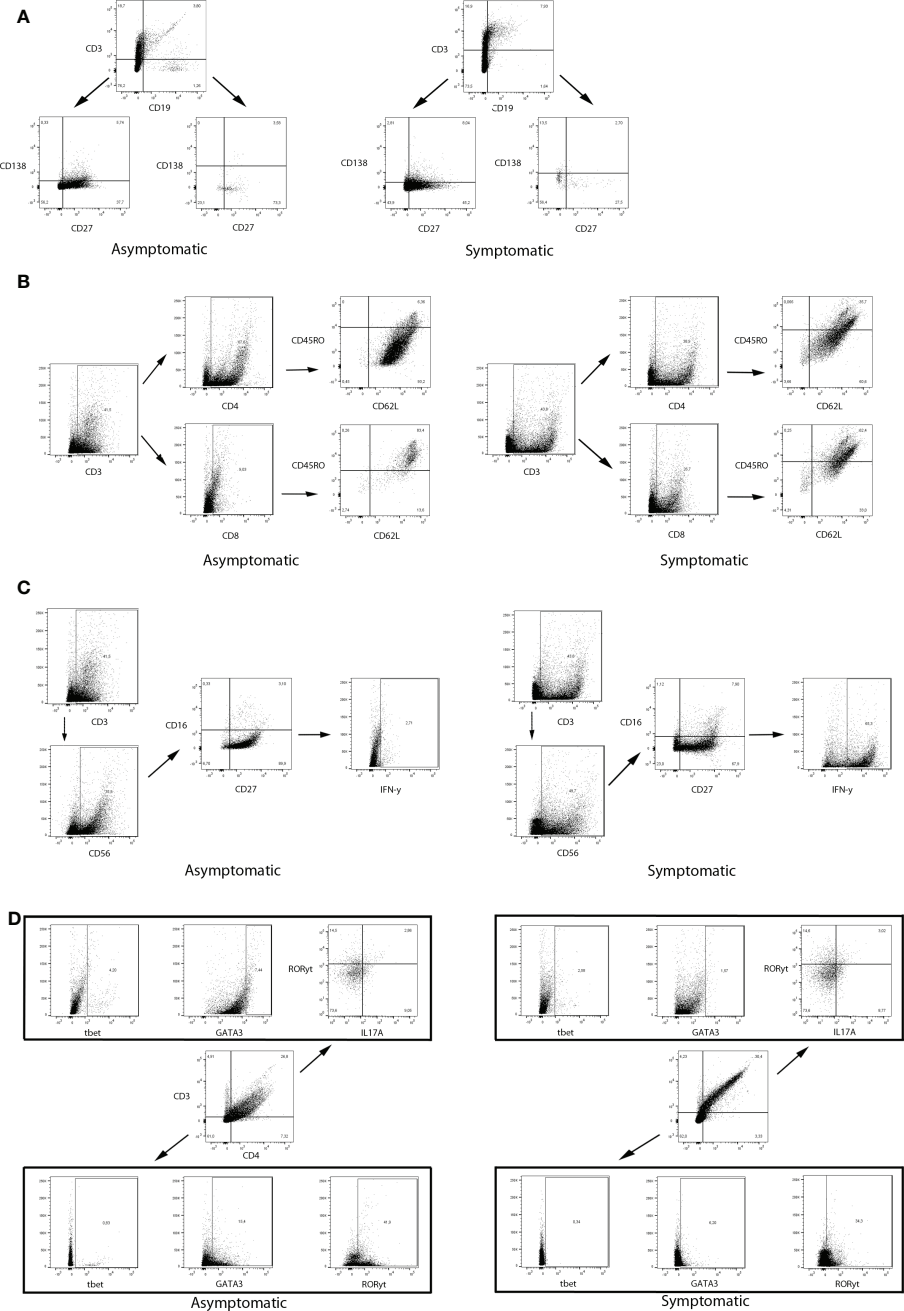
Gate strategies and representative dot plot graphs from flow cytometry data of two women (Asymptomatic number 7 and Symptomatic number 4). **(A)** CD3^-^CD19^-^ and CD3^-^CD19^+^ cells were gated for CD138 and CD27 analyzis. **(B)** CD3^+^ cells were further evaluated for CD4 or CD8. CD3^+^CD4^+^ and CD3^+^CD8^+^ cells were gated for CD45RO and CD62L analyzis. **(C)** CD3^-^ cells were evaluated for CD56. Both CD56^bright^ and CD56^dim^ were further analyzed for CD16 and CD27 expression. CD3^-^CD56^bright^CD16^-^CD27^+^ cells were evaluated for IFN-γ analyzis. **(D)** CD3^+^CD4^+^ and CD3^-^CD4^-^ were evaluated for tbet, GATA3, and RORγt. CD3^+^CD4^+^RORγt^+^ were also analyzed for IL17A.

Regarding the B lymphocytes and plasm cell populations, CD3-CD19^-^CD138^+^CD27^++^, CD3^-^CD19^+^CD138^-^CD27^-^, and CD3^-^CD19^+^CD138^-^CD27^+^ cells were found in both studied groups, although no significant changes were observed (**Figures 4C–E**). The same unchanged pattern was evidenced in both naive T cells, CD3^+^CD4^+^CD45RO^-^CD62L^+^, and CD3^+^CD8^+^CD45RO^-^CD62L^+^, and in all T cell memory populations: CD3^+^CD4^+^CD45RO^+^CD62L^+^, CD3^+^CD8^+^CD45RO^+^CD62L^+^, CD3^+^CD4^+^CD45RO^+^CD62L^-^, CD3^+^CD8^+^CD45RO^+^CD62L^-^ (**Figures 4F–K**). Effector T cells and different Th cell populations, such as CD3^+^CD4^+^CD45RO^-^CD62L^-^, CD3^+^CD8^+^CD45RO^-^CD62L^-^, CD3^+^CD4^+^Tbet^+^, CD3^+^CD4^+^GATA3^+^, and CD3^+^CD4^+^RORγt^+^IL17A^+^ cells were present in all samples, but unchanged between the studied groups (**Figures 4L–P**). The innate lymphoid cells (ILC) population CD3^-^CD4^-^Tbet^+^, CD3^-^CD4^-^RORγt^+^, and CD3^-^CD4^-^GATA3^+^, were also analysed, but no changes were observed in both groups (**Figures 4Q–S**). Notably, two different populations of NK cells were greatly increased in the symptomatic group compared to the asymptomatic group. As such, CD3^-^CD56^bright^CD16^-^CD27^+^IFN-γ^+^ cells changed from 0.08 ± 0.04% in the asymptomatic group to 6.5 ± 1.1% in the symptomatic group (*p* < 0.0001) (**Figure 4T**), whereas CD3^-^CD56^dim^CD16^+^CD27^-^ cells increased from 0.26 ± 0.11% in the asymptomatic group to 0.82 ± 0.22% in the symptomatic group (*p* = 0.0093) (**Figure 4U**). Gate strategies and representative images are depicted in **Figure 5**.

## Discussion

Despite the magnitude of COVID-19 and its impacts on health, few reports are available on the composition of colostrum after women were stricken with this disease during pregnancy, either with or without symptoms (31, 32).

A study conducted by Sánchez-Garcia et al. (2021) (33) in Madrid, Spain, addressed the prevalence of SARS-CoV-2 and the evolutionary profile of immune compounds in breastmilk of positive mothers, according to time and disease state. It involved 37 women in the study group and 45 in the control group. The investigation revealed that the colostrum of mothers infected with the virus, with samples collected every 72 h from delivery, had a distinct immunological profile. On the other hand, there was a reduction in these compounds in women in the control group, suggesting that further studies should be carried out to assess whether such findings indicate an efficient reaction of the organism against SARS-CoV-2 infection, aiming at protecting the child (33).

Few studies evaluated oxidative stress in colostrum, especially in cases of COVID-19. To provide protection, breast milk adapts to the child’s needs, its composition modifying depending on several factors. Therefore, it is a food extremely rich in biologically active ingredients, such as enzymes, vitamins, and proteins, being a powerful antioxidant that protects the child against diseases (34). Breast milk provides nutrients for the proper growth and development of the child. Its content is variable and dependent on factors such as body composition, environment, climate, ethnicity, diet, maternal age and gestational age, with the last two variables being important in the concentration of bioactive molecules (35). The enzymatic and non-enzymatic antioxidants that occur naturally in breast milk support the neonate’s antioxidant defences against infection and disease. The enzymatic antioxidant system includes GPx, GR, glutathione S-transferase (GST), SOD and CAT. These systems scavenge radicals and peroxides and prevent their formation (36). One study by Zarban et al. (2009) (37) in milk samples (colostrum, transition milk, and mature milk) from 115 healthy women and full-term newborns showed that the total antioxidant status was higher in colostrum than in transition milk and mature milk. These data suggest that colostrum consumption in the first days of life is vital, due to its high antioxidant potential. In addition, this food is also rich in nutrients, as well as in immunological components, which confer numerous benefits to the health of newborns.

In colostrum, a higher total antioxidant status is identified, compared to mature milk, and its radical scavenging activity decreases during lactation. Thus, breastfed children demonstrate a more efficient antioxidant barrier, suggesting, as in the present study, that the maternal organism adapts and prioritizes the child’s health, producing and secreting a greater amount of antioxidant in those affected by the disease. That is, a compensatory adaptation to oxidative stress occurs to restore redox balance (38).

The findings of the present study were related to an increase in GSH and a decrease in H_2_O_2_ in the colostrum of symptomatic women by COVID-19, with no differences between GPx levels. Although there was no statistically significant difference between the groups, there was still a trend towards a reduction in GSSG in the symptomatic group. GSH is the most abundant thiol in mammals, being essential in the defence of cells and tissues against oxidative stress. It participates in numerous cellular reactions, directly and indirectly to scavenge free radicals and other reactive oxygen species (such as H_2_O_2_, for example), where it is oxidized to form GSSG, which is then reduced to GSH by NADPH-dependent glutathione reductase (39, 40). GPx inhibits the lipid peroxidation process. H_2_O_2_, derived from the catalytic reduction of the radical anion superoxide (O_2_^•-^), can be produced by mitochondria and is found in breast milk. It plays several biological cell-signalling roles. Its elimination occurs initially by CAT, very effectively, and by GPx. However, in the context of oxidative stress, increased levels of this species can be found (41).

Despite the absence of the quantification of CAT, the present results can suggest that symptomatic women were able to offer greater antioxidant protection to the neonates. It is known that cold storage can significantly reduce the antioxidant capacity of breast milk (42, 43), however our analyzes were standardized initially, aiming at preserving the immunological properties of breast milk (22). Additionally, few studies have analyzed markers of oxidative stress in human colostrum (44–46), and to the best of our knowledge, none determined the shelf life of CAT, after different storage periods at -80 °C. In this sense, our research demonstrates that, unlike other antioxidant enzymes, CAT in biological media seems to be more sensitive to freezing. This finding reinforces the need for future studies to investigate the stability of CAT in human colostrum under different conditions of time and temperature, in order to ensure a better supply of bioactive immunological components, such as antioxidant enzymes.

Furthermore, studies confirm higher concentrations of immunological components, growth factors, bioactive compounds, cytokines, and chemokines in colostrum compared to mature milk, even in women without infections and/or complications during pregnancy and postpartum (5, 47). Moreover, changes in the composition of breast milk were observed according to the conditions of the newborn, including the increases in total number of leukocytes in human milk, especially colostrum, in relation to the infants without infection, in diseases such as SARS-CoV-2. Such alterations may reflect changes in the cytokine profile observed in the presence of the disease, with increased levels in the colostrum of those infected women. Thus, the infant’s immune system can be stimulated from this colostrum and increases the production of defence cells to maintain an organic balance in these individuals. Therefore, it is suggested that changes in the immunological profile of human milk is a form of adaptation to provide additional protection to the child, with a permanent dynamic interaction between the mother-child dyad (33, 48).

Cytokines in human milk may have an immunostimulatory or immunomodulatory effect on phagocytic cells and on lymphocytes involved in developing the specific immune responses of the child and acting in the prevention of allergies and hypersensitivities (49). They are suggested to be secreted by immune cells present in the mammary glands, although the exact sources remain to be unveiled (50). It is also unknown which cytokines survive the passage through the infant stomach, but some data suggest that cytokines remain protected until they reach the intestine (51). Moreover, these cytokines can act on cells of breast milk itself, stimulating the innate and acquired immune response, including growth, differentiation, and production of antibodies by B cells (52), and they help breast milk derived cells to surpass infant mucosal epithelial barriers (53).

The study from Narayanaswamy and collaborators (2021) (15) was the first to show a cytokine panel from the colostrum of women with COVID-19, comparing asymptomatic and symptomatic women. They have described that the levels of IFN-γ, IL-4, IL-6, and IL-12 were significantly higher in the colostrum from the symptomatic group, although their possible functions and the neonatal repercussions were not deeply explored. Differently, our study is the first to describe the cytokines alterations in the colostrum after asymptomatic and symptomatic COVID-19 in pregnancy. As such, only two cytokines were different in the symptomatic group that had a reduction in GM-CSF and IFN-α levels.

In the breast milk, GM-CSF is known to be produced by macrophages and acts autocrinally and synergistically with IL-4 to differentiate into CD1^+^ dendritic cells (54). It can also increase phagocytosis and pathogen killing by macrophages, inhibiting *Staphylococcus aureus* infections in the mammary gland (55), and up-modulate *Toll*-like receptor (TLR)-mediated responses (56, 57). Other known producers are NK cells, as GM-CSF can also be important to restoration of immune functions of tolerant leukocytes under inflammatory events (58). Regarding IFN-α, despite the antiviral role, this cytokine is involved in the activation of innate and adaptive immune cells responses and enhances NK cells cytotoxic capacity (59). In a case report published by Yu and collaborators (2021) (60), they showed an eightfold increase in IFN-α^+^ leukocytes from the mature milk of women with COVID-19 infection.

Interestingly, IFN-α and GM-CSF are needed to differentiate monocytes into IFN-producing plasmacytoid dendritic cells (pDC) (61), which not only stimulates adaptive, but also mediates, innate immune responses with the production of large amounts of IFN-I (62, 63). Rather frequent in the gut, pDC are thought to play important roles in maintaining intestinal homeostasis (64), but also capable of activating Th17 cell development and inducing inflammation (62).

Our findings indicate the presence of specific IgA for SARS-CoV-2 in both groups, suggesting a previous infection promoting a humoral response; however, no significant differences were found between the groups. IgA antibodies coat the gastrointestinal and respiratory mucosa, where they block the entry of foreign antigens and viruses, providing a protective action especially for infectious diseases, so their presence in the colostrum of mothers who were infected by the virus seems to be a protective mechanism for the child (65). Corroborating our findings, Pace et al. (2021) (13) identified that 76% of mature milk samples from COVID-19 infected women admitted to their study contained IgA specific to SARS-CoV-2. Yet, similarly, colostrum samples from women who were positive for SARS-CoV-2 had 73% IgA reactivity (15).

Since several microorganisms use the mucosae as a portal of entry, neonates have increased risk for infections (66). During this critical period of vulnerability, neonate defences rely on breast milk protective factors, and the development of their own immune system increased by the breast milk, as well (67). These characteristics reinforce the idea that the breast milk provides a highly immunoactive system primed to protect from potential pathogens the mother-infant dyad may encounter (68). As such, the immune cells play a pivotal role in bridging the gap between birth and the child’s development of a fully functional immune system (68). Nevertheless, studies that observe cell transfer from the breast milk to neonate tissues have obvious limitations (69, 70). The breast milk-neonate cell transfer, a case of microchimerism, is thought to heavily influence the neonate immune system development, inducing neonatal tolerance to non-inherited maternal antigens, and fast responding against novel antigens (71).

The colostrum immune cell frequency during an infection is dynamic, dramatically increasing in comparison to non-infected women, and after the infection returning to normal levels (71). In our results, possible differences comparing different colostrum lymphoid cell populations after asymptomatic or symptomatic COVID-19 in pregnancy were, pioneeringly, analysed herein, differing from the literature. Firstly, total cell count in both analysed groups were compatible with the literature, as well as all the encountered cell types (49). It is known that apart from epithelial cells, macrophages, and neutrophils, lymphoid cells are one of the most important populations present in the colostrum (49, 68). Despite cellular frequency importance, multiple maternal characteristics can influence the cellular composition of the colostrum, such as diet and age, which could derive in high variable results in different populations (72, 73).

Usually, most of the lymphoid cells are CD3^+^ T cells, which are known to derive from mucosal sites rather than from the blood (68). The breast milk CD3^+^ CD4^+^ T cells are, commonly, activated with the expression of CD45RO, a surface receptor associated with memory. The CD3^+^ CD4^+^ T cells transfer contributes to the modulation of immune responses, possibly allowing a major repertoire of T cell functions in the neonate (69). Moreover, breast milk CD3^+^ T cells are equally distributed between CD4^+^ and CD8^+ 75^. Conversely, both groups analysed herein were different, as CD4^+^ and CD8^+^ T cells were not equally distributed, but at the respective proportion of 3:1. Since COVID-19 is known to selectively and systemically reduce CD8^+^ T cells (74), the colostrum amount of these cells could be impaired in both groups. Another hypothesis relies on the fact that our population might have a different basal proportion of these cells in the colostrum, as frequency differences could differ depending on the studied population (72, 73, 75), and even from study differences regarding the type of breast milk analysed (colostrum, transitional milk, or mature milk).

At birth, cells of the innate immune system, IgM, and IgG^+^ B cells are present in the neonate intestinal mucosae, but IgA^+^ B cells are rare. These B cells are known to be majority CD27^+^ memory cells, primed to secrete antibodies; as well as plasma cells (76), which are thought to infiltrate several neonate tissues and survive until adulthood (69). Adversely, in our samples we have a similar proportion of naïve CD27^-^ B cells, memory CD27^+^ B cells, and plasma cells. Our results also showed a great amount of different ILCs, including NK cells. These cells regulate homeostasis, inflammation, and they have antimicrobial functions. Usually in the breast milk, ILC1 is more frequent than the other subtypes, and in rodents, they can migrate to different parts of the pups, such as intestines, lungs, heart, and thymus (69). The NK cells can be divided in two subpopulations, CD56^bright^ CD16^-^ and CD56^dim^ CD16^+^ cells with predominant cytokine production or cytotoxic functions, respectively (77). Unprecedentedly, our results depict both subtypes to be greatly increased in the symptomatic group, which could implicate an increased transfer of these cells to infant’s intestines. Generally, lymphoid cells expressing CD56 isolated from the gut do not express CD16, being considered lymphoid effectors that have a relevant role in the regulation of gut homeostasis (78), whereas CD56^dim^ CD16^+^ cells are involved in the fast response to viral infections (79). As such, the increase in both NK cells populations could be, absolutely, interesting to the increase of the infant gut innate immunity and homeostasis maintenance. Altogether, it is possible that reduced GM-CSF and IFN-α could lead to reduced maternal pDC activity, which is important to differentiate T cells in a Th17 phenotype after TLR activation (80), indicating a slightly reduced inflammatory potential in the neonates. Moreover, the NK cells increase could enhance neonatal antiviral innate response, as well as to maintain gut homeostasis. Although interesting hypothesis, further studies are needed to correlate our findings with the outcomes potentially involved with the maternal-infant dyad.

When interpreting our findings, three limitations must be considered. The first concerns the lack of a control group composed of healthy lactating women with no history of Covid-19 to compare our findings. However, it was not possible to locate pre-pandemic colostrum samples. The second limitation concerns the small sample size, possibly limiting the power to detect other differences between the groups. However, due to the various restrictions and social isolation imposed by the pandemic and the beginning of pregnant women’s vaccination, it was impossible to extend the collections. Finally, due to the lack of studies reporting the valuable lifetime of the CAT in human colostrum, our analyses were standardized and performed within ten months after collection, making its determination unfeasible. However, this limitation provides a new look at the impact of freezing on the antioxidant capacity of colostrum, particularly regarding CAT activity. As strengths of the study, we highlight that up to our knowledge; the present work is one of the few that analyzed a wide variety of components, especially immunophenotyping and oxidative stress markers after COVID-19 in pregnancy. Additionally, few studies concerning breast milk were able to explore the colostrum that is produced in small amounts, turning its collection difficult.

## Conclusions

Our study is among the first to demonstrate the impact of symptomatic and asymptomatic COVID-19 infection during pregnancy on the composition of different bioactive colostrum components. In a nutshell, an increase in total GSH levels and reduced levels of H_2_O_2_, IFN-α2, and GM-CSF have been observed in the colostrum of the symptomatic group, and a considerable increase in both NK cell subtypes.

These results demonstrate a greater antioxidant activity in the colostrum of women, symptomatic for COVID-19, during pregnancy, in addition to changes in the immunological composition that may represent an increase in the infant’s intestinal innate immunity and maintenance of homeostasis. Despite the absence of a healthy control group with no history of COVID-19 during pregnancy, our findings suggest a mechanism of adaptation of the organism against mild and symptomatic SARS-CoV 2 infection, and demonstrates how adaptive is colostrum composition, after infection. In this way, the encouragement of early breastfeeding in women, who have been affected by COVID-19 during pregnancy, is highlighted, to ensure greater protection for the conceptus. The lack of a control group and the small number of subjects may affect the relationship between the analyzed immunological markers. Thus, further studies are needed to establish the influence of viral infections on colostrum composition, as well as investigations in samples of colostrum and mature breast milk from lactating women vaccinated against COVID-19.

## Data availability statement

The raw data supporting the conclusions of this article will be made available by the authors, without undue reservation.

## Ethics statement

The studies involving human participants were reviewed and approved by The Ethical Committee of Unversidade Federal de Alagoas (UFAL) approved the study for human subjects (n. 36006920.8.0000.5013), and all women signed a free and informed consent. The recruitment occurred in three hospitals in Maceio-Alagoas, Brazil: University Hospital Professor Alberto Antunes (HUPAA), Santa Monica Maternity School (MESM), and Santo Antonio General Hospital. Written informed consent from the participants’ legal guardian/next of kin was not required to participate in this study in accordance with the national legislation and the institutional requirements.

## Author contributions

Conceptualization: MG, FM, AB, AO. Validation: MG, FM, AB, AO. Formal analysis, investigation: NG, RB, ET, KB. Writing—original draft preparation: NG, MT, MF, FM. Writing—review and editing: MG. Visualization: MG. Supervision: MG, AO, AB. All authors have read and agreed to the published version of the manuscript. All authors contributed to the article and approved the submitted version.
